# Digital twin simulations of theory-driven crisis messaging during hurricane evacuations in synthetic populations: a Miami-Dade County case study

**DOI:** 10.3389/frai.2026.1715883

**Published:** 2026-02-11

**Authors:** Brandon Walling, Linda Desens, Vanessa Howard, Rhys O’Neill, Denise Scannell, Mary Giammarino, Sara Beth Elson, Scott Rosen

**Affiliations:** The MITRE Corporation, McLean, VA, United States

**Keywords:** agentic AI, artificial intelligence, autonomous agents, behavioral science theories, crisis communication, crisis management, digital twins, evacuation

## Abstract

**Background:**

Digital twin and agentic artificial intelligence technology provide innovative systems for testing behavioral science theory, which can improve emergency communication in crisis situations. More advanced and effective evidence-based messaging is needed for better safety preparation for extreme weather and more trusted evacuation communication.

**Methods:**

This study developed a digital twin of Miami-Dade County populated with a synthetic population embedded with behavioral theory (Extended Parallel Process Model, Theory of Planned Behavior) and the development of a Message Assessment Framework (MAF) to systematically test theory-based crisis messages. Agents were exposed to fear-only, efficacy-only, norm-only, combined fear+efficacy, combined fear+efficacy+norm, and a neutral control message.

**Results:**

Messages grounded in behavioral theory were more effective than the control message at encouraging evacuation. Messages that combined fear and efficacy provided the best results in the synthetic population’s decision to evacuate (OR = 15.45, *p* < 0.001), while adding social cues did not produce a statistically distinguishable added benefit.

**Discussion:**

This research demonstrates a proof-of-concept approach for using agentic AI and digital twins to pre-test communication strategies, offering a scalable method for optimizing emergency messaging prior to real-world implementation.

## 1 Introduction

Each year, coastal U.S. communities face storms that require fast and sometimes difficult evacuation decisions. Even with better forecasts and preparedness since Hurricane Katrina in 2005, many residents delay or refuse to evacuate when ordered (Logan et al., 2022). Recent research has shown that evacuation compliance in crisis response could be improved by providing communities with timelier information and support to evacuate via improved communication strategies (Logan et al., 2022; Shultz et al., 2020).

Evacuations function as a core protective measure for communities to prevent injury and death in hurricane situations. Crisis communication research shows that people are more likely to take protective action when messages combine information about the threat with clear steps they can take and social cues that support action so that they have confidence in their ability to safely evacuate from their homes (Morss et al., 2024). Yet, most existing approaches to crisis response messaging are intuition-based, relying on expert judgment, anecdotal evidence, or historical precedent rather than systematic, data-driven, theory-based experimentation. Intuition-based approaches, while sometimes valuable in urgent and desperate situations, often fail to account for the wide-ranging and ever-adapting responses of heterogenous populations (Edwards, 2021; Taylor et al., 2020; Taylor et al., 2023), especially in urban environments.

The Extended Parallel Process Model (EPPM) and the Theory of Planned Behavior (TPB) both highlight the importance of fear, efficacy, and social norms in shaping behavior (Ajzen, 2020; Tannenbaum et al., 2015; Witte and Allen, 2000). Past studies have shown that fear and efficacy appeals can improve compliance in surveys and field experiments (Ort and Fahr, 2018; Siegenthaler et al., 2021). What is unclear is how these strategies work in more complex settings that mimic real-world conditions.

The purpose of this research is to test how different theory-based message framings, namely fear only, efficacy only, norm only, combined fear + efficacy, and combined fear + efficacy + norm, affect evacuation behavior in a simulated environment. This study makes a methodological contribution towards accurately modeling realistic behavior by developing a digital twin of Miami-Dade County, populated with behavioral theory-based agents built from real demographic and environmental data. This approach allowed us to compare theory-based messaging strategies under controlled and repeatable conditions.

Our research makes the following contributions through the development and experimentation of a digital twin:

1. *Demonstrates a new use of behavioral theory within an agent-based AI simulation*, showing how EPPM and TPB constructs can be tested in a digital twin environment to evaluate crisis messaging strategies.
2. *Introduces and applies a Message Assessment Framework (MAF)* as a systematic tool to measure the impact of theory-based message framings on evacuation behavior in simulated populations.

A hurricane scenario was selected as the case for this research. Most evacuation messaging still relies on expert judgment and past experience instead of systematic, data-driven testing (Taylor et al., 2020; Taylor et al., 2023). Recent studies suggest digital twins and agent-based simulations as promising tools to address this gap (Botín-Sanabria et al., 2022; Pretel et al., 2024). These methods make it possible to test different messages with synthetic populations built from real-world data, offering insights that can improve crisis communication strategies in the real world.

## 2 Literature summary

### 2.1 Modeling human behavior in crisis scenarios with digital twins and agentic AI

Crisis simulations involving autonomous AI agents embedded in digital twins can advance emergency preparedness and crisis communication by giving decision makers a dynamic, data-driven reflection of the real world (Chapuis et al., 2022). They function as a virtual testing ground with bidirectional data (near real-time data, sensor inputs, and behavioral models) flowing into and out of a virtual twin of a physical place, such as a county or other region, to inform real-life decision-making (Botín-Sanabria et al., 2022; Ghaffarian, 2025; Riaz et al., 2023; Wright and Davidson, 2020). They can simulate and test variables such as interventions, policies, and messaging before implementation in the real world, saving time, money, and sometimes even lives (Penverne et al., 2024). In contrast, current models are only able to analyze historical data and lack the ability to simulate a system’s current state (Penverne et al., 2024).

Digital twins provide an opportunity to improve hurricane response planning and message testing strategies with a human-in-the-loop to interpret and focus the simulation despite the chaotic and quickly evolving environment of a hurricane (Ghaffarian, 2025). Coupled with agent-based modeling, which is meant to help create synthetic populations that are representative of the real world in an accurate way, digital twins allow for dynamic message-testing with embedded behavioral theories like EPPM or TPB, for example, to capture heterogeneous behaviors like evacuation compliance, panic, or general sentiment (Bilal et al., 2025; Croatti et al., 2020; Pretel et al., 2024). These scenarios can inform the timing or phasing of messages to at-risk populations, traffic control, shelter strategy, or other common barriers or concerns (Bilal et al., 2025; Botín-Sanabria et al., 2022).

Agentic AI represents a paradigm shift in the simulation of human behavior, especially in crisis scenarios where understanding rapid, adaptive decision-making is essential. Unlike traditional agent-based models that rely on static rule sets, agentic AI systems are designed to emulate autonomous, goal-driven entities capable of reasoning, learning, and adapting within dynamic environments (Acharya et al., 2025; Pretel et al., 2024). The agents within the system can be trained to integrate internal cognitive and emotional states such as fear, trust, and efficacy grounded in behavioral theory, enabling them to process information, evaluate risk, and update their beliefs and behavior in response to an evolving environment around them.

Agentic AI technology has been used alongside digital twins to create models that predict human behavior in different complex scenarios that span industrial production and engineering, automotive, logistics, and healthcare industries (Botín-Sanabria et al., 2022; Galuzin et al., 2022; Pang et al., 2021). Insights from applications using multi-agent systems and digital twins are data-driven and empirically testable, making this approach well-suited for exploratory or preliminary research, such as message testing. These applications provide a controlled and risk-free environment to inform study design. (Botín-Sanabria et al., 2022; Dembski et al., 2020; Pretel et al., 2024). By developing well-trained agentic AI systems, researchers can simulate human behavior to observe how varied population segments might respond to different messages or crisis response strategies (Collins et al., 2018).

Agentic AI has been used to create virtual replicas of disaster management and crisis communication scenarios, modeling behavior of specific population segments under varying conditions (Acharya et al., 2025; Galuzin et al., 2022; Pretel et al., 2024). For example, Pang et al. (2021) used a system of collaborative city digital twins (multiple digital twins that worked with and learned from each other) to simulate a federated learning solution to respond to the COVID-19 crisis (Pang et al., 2021). In this study, agentic AI and digital twins simulated responses to the health crisis to plan resource management across different cities within a targeted region, considering city-specific population characteristics to improve the global response across all cities. Another example that sets precedence for these concepts is the CitySEIRCast simulation tool, which is an agent-based city digital twin focusing on COVID-19 pandemic analysis (Bilal et al., 2025). The tool combined the virtual environment (digital twin) of a physical city with a synthetic population (agents) and disease model that could interact and help inform decision-making by performing various what-if scenarios meant to understand likely responses, actions, and disease transmission. This is a relevant comparison to hurricane events, as they both produce quickly changing environments that require calculated, sensitive responses to keep people safe and provide them with the right information at the right time.

Although powerful, the simulations and agent behavior are only as good as the data and training that go into the models. The development of accurate AI systems requires extensive data pre-processing, thorough data pipelines, and high-quality training to ensure realistic output (Acharya et al., 2025; Pretel et al., 2024). Human oversight and feedback loops are also critical to ensure that outputs are reliable and valid. Agentic AI provides a new method for simulation under controlled conditions to test theory and predict behavior, enabling researchers to systematically evaluate how various populations might respond to different message strategies, but the predictive power of these models is only as strong as the realism of the agents themselves. The next step in the evolution of digital twins is to ensure that the synthetic populations are designed in a way that mirrors the complexities of the real-world populations that they are meant to represent, which enables simulations that are both theoretically grounded and practically relevant.

### 2.2 Integration of behavioral theory to design realistic populations for simulation

To fully realize the potential of digital twin simulation, it is not enough to replicate demographic or environmental features, the behavioral realism of the agents must be addressed. Earlier crisis simulations often relied on agent-based models (ABMs) that represented agents as rule-based entities designed to achieve specific goals. Research has demonstrated the value of including behavioral theories into agent design, for example, combining multiple behavioral theories such as the Health Belief Model, goal framing theory, and the Theory of Planned Behavior in a health-seeking model improves predictive accuracy by 15–30% when compared to single theory models, while capturing complex temporal and demographic patterns (Taghikhah et al., 2025).

Studies that applied EPPM in public health contexts demonstrated that the EPPM constructs such as susceptibility, efficacy, and perceived severity are strong predictors of protective behavior. One study showed that these factors predicted close to 50% of people’s engagement in preventive behaviors while fear control had a negative effect (Naroozi Masir et al., 2023). These studies support the improved ability for agents to mimic human response to crisis messages when behavioral theory constructs are embedded in their design.

Although behavioral theory can help explain the way individuals handle hurricane risk and evacuation decision-making, many studies only focus on demographic and contextual predictors such as age, hazard proximity, or income instead of applying and integrating behavioral theories into their research as well. Studies of predictive evacuation models will reference frameworks (for example, EPPM, the Protective Action Decision Model, and TPB) but they are not often operationalized within the study (Adjei et al., 2022; Wang et al., 2021).

### 2.3 Behavioral theories for agent reasoning and message framing

In the previous section, we introduced the importance of including behavioral theories in the design of autonomous agents to create realistic, synthetic environments. In this section, we will discuss the application of behavioral models as the foundation for understanding how individuals might interpret and respond to emergency messaging and associated communication by applying two relevant and complementary frameworks, EPPM and TPB.

#### 2.3.1 Extended parallel process model

EPPM is a theoretical framework that is not only widely used in health communication but also across social and behavior change communication, such as crisis and disaster messaging. EPPM constructs are used to frame risk, bolster perceived efficacy, and motivate protective action in contexts such as pandemics and other emergencies (Munro et al., 2025; Tsoy, 2022; Witte and Allen, 2000).

EPPM emphasizes the role of two thought processes that explain human response to risk messages: the process of threat detection and the process of perceived efficacy. The threat detection process takes place when an individual perceives that there is a risk of danger. The efficacy process involves an individual’s perception of their ability to respond and the likelihood of their response alleviating the threat. According to EPPM, when perceived threat and perceived efficacy are high, individuals are motivated to engage in the danger control process because they believe the danger is both serious and manageable, which leads to taking action to protect themselves from danger, like evacuating during a hurricane warning. However, when perceived threat is high but efficacy is low because individuals either think there is nothing they can do about the threat or that their potential actions would not be effective in alleviating it, they are more likely to engage in “fear control” responses, such as denial, avoidance, becoming defensive about the risk, or even panic (Witte, 1992; Witte and Allen, 2000).

Meta-analytic evidence over the past several decades has supported the effectiveness of using fear appeals to motivate behavior change against risk, but only when the threat information is paired with clear efficacy cues with achievable guidance about how to effectively alleviate the threat (Tannenbaum et al., 2015; Witte and Allen, 2000). Combined messages that integrate both threat and efficacy elements have been shown to be most effective in promoting adaptive behavior (Bigsby and Albarracin, 2022; Ort and Fahr, 2018). Recent studies have extended EPPM to a wide range of crisis scenarios, including public health outbreaks (Islam et al., 2023), vaccination campaigns (Ort and Fahr, 2018), and disaster alert systems (Woo Yoo et al., 2021). These applications emphasize the importance of tailoring messages to recipients’ cognitive and emotional states, as well as the need for ongoing refinement of theoretical models to account for emerging communication channels and sociocultural factors.

Despite the research applying EPPM to various contexts, public alerts often highlight risk while leaving out specific instructions—like transportation options, shelter access, or other concrete resources—which undermines compliance (Seeger, 2006; Vos and Buckner, 2016). In the context of crisis communication, EPPM provides a structured approach for simulation work, offering parameters to design and test message variants that frame risk and efficacy in different ways to influence the likelihood of compliance. For example, fear-based messages may highlight the imminent danger of an incoming hurricane, while efficacy-based messages focus on the availability of shelters and evacuation routes to reduce risk. Combined messages balance these elements, aiming to maximize both perceived threat and efficacy to elicit the desired behavioral response. The present study operationalizes these constructs within a digital twin environment, enabling systematic evaluation of their impact on evacuation compliance among synthetic agents.

#### 2.3.2 Theory of planned behavior

TPB is another well-known behavioral theory that takes a different angle, focusing on how intentions shape behavior (Ajzen, 2020). TPB focuses on behavioral intention and predicts how individuals decide on an action based on perceived social norms, self-efficacy, and attitudes (Ajzen, 2020). Three psychological components influence this decision. This includes an individual’s attitude toward the behavior, such as how a person evaluates the consequences of taking a particular action. Additionally, subjective norms, or the social pressure from peers, family members, or even authoritative figures, can influence intentions to behave in certain ways (Collins et al., 2018). For example, in the case of a flood, a person will evacuate if they perceive that others around them, such as their neighborhood or community, expect them to evacuate (Rufat et al., 2024). The third psychological component is perceived behavioral control, where an individual perceives how easy or difficult it would be for them to perform a certain behavior or action. This includes anything that influences their belief in their ability to prepare for disasters, such as access to information, transportation, and shelter (Tabatabaei et al., 2025). The theory has been shown to predict behavior across a wide range of health and safety contexts (Armitage and Conner, 2001; Srivastava et al., 2023).

Prior research has demonstrated the positive impact between the subjective norm construct of TPB and disaster preparedness behavior, where individuals were more likely to take disaster preparedness actions if they felt that those actions were approved by family members, friends, significant others, and health professionals (Tabatabaei et al., 2025). Subjective norms have been shown to be important factors in influencing individuals to store household emergency supplies in the context of a public health emergency (Wang et al., 2023).

We were interested in understanding how synthetic agents would respond to the subjective norm construct in messaging and, most importantly, whether it effectively drives agent behaviors to evacuate. By embedding TPB constructs within agents, we can produce behaviors that enact the theoretical constructs in varying degrees.

EPPM and TPB each offer useful, but different, ways to understand how people make decisions in a crisis. EPPM looks at how people respond emotionally and cognitively to threats, especially the role of fear and how confident they feel in acting. TPB, on the other hand, focuses more on how social norms, attitudes, and perceived control shape behavior over time. By combining constructs from both models—like fear, efficacy, and social pressure, we can better understand what drives behavior and test how different messages might work in different communities (Kim et al., 2024).

Using both theoretical models together gives us a more complete picture. EPPM helps explain immediate reactions to crisis messaging, while TPB captures the social and psychological factors that shape longer-term intentions. When these frameworks are built into digital twin simulations, it becomes possible to test different communication strategies in realistic, controlled environments. Decision-makers can leverage this capability to select and design effective messaging prior to implementing it in the real world.

### 2.4 Gaps in literature and research objective

While existing crisis communications research has tested the effects of message framing using surveys, field studies, and laboratory experiments, most studies focus on single message elements in isolation and lack a systematic way to compare multiple framings under controlled but realistic conditions. Previous approaches have shown that fear, efficacy, and social norms play important roles in shaping protective behaviors (Tannenbaum et al., 2015; Siegenthaler et al., 2021; Wong-Parodi and Garfin, 2022) but provide limited guidance for evaluating message effectiveness in complex settings such as evacuations, where multiple behavioral constructs may interact.

**Figure 1:**
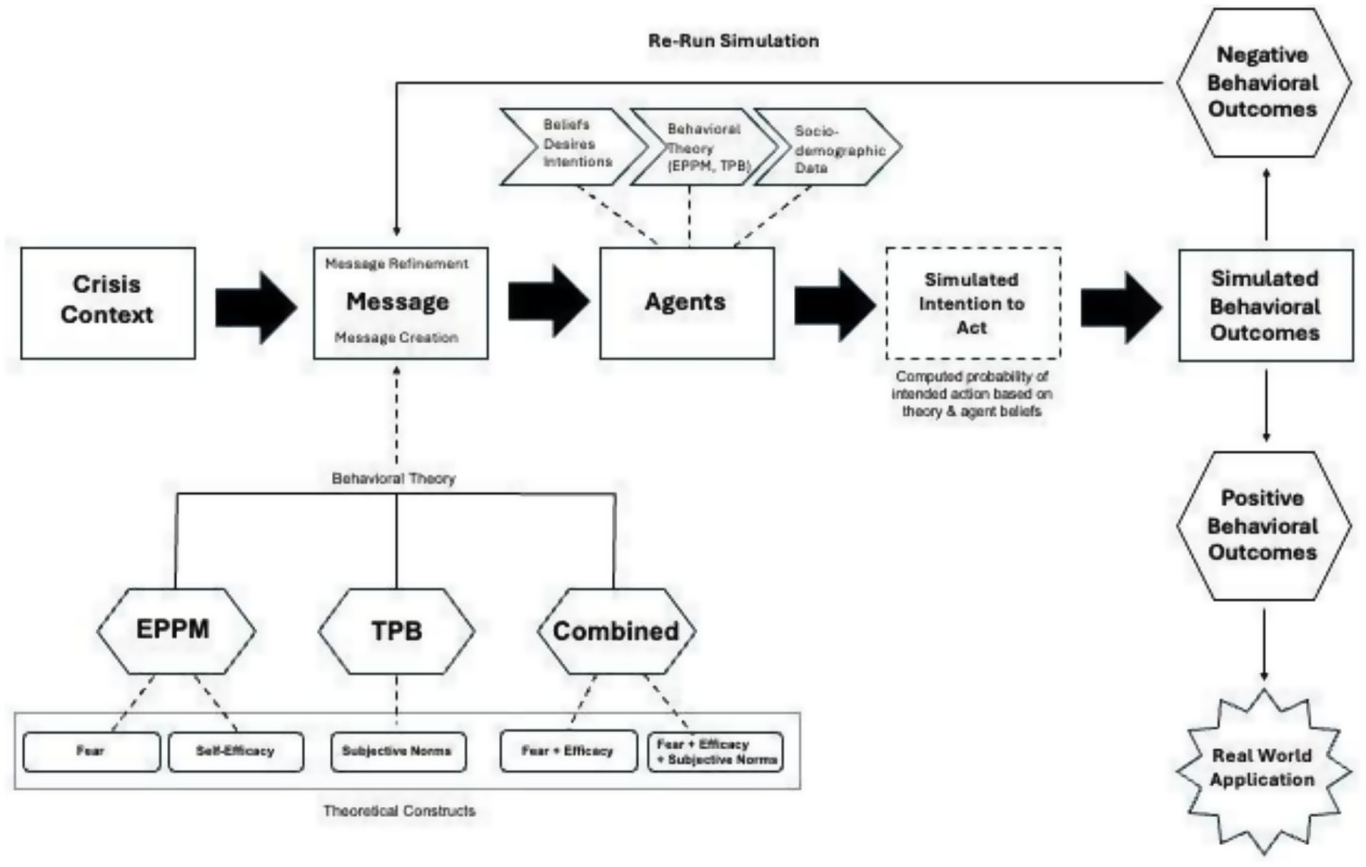
Message assessment framework (MAF). This framework integrates constructs from EPPM (threat and efficacy), TPB (subjective norms and perceived behavioral control), and BDI architecture to connect theory-based message framing with agent decision processes.

This gap highlights the need for a structured, theory-driven framework that can be applied within simulations to assess how different message framings influence protective decision-making. To address this, the present study introduces a Message Assessment Framework (MAF) (Figure 1) that integrates constructs from EPPM and TPB and applies them in a digital twin environment. The MAF provided a consistent way to classify messages by their emphasis on threat, efficacy, and social norms, both individually and in combination. Embedding this framework within the digital twin allowed for controlled, theory-driven comparisons across message framings and represents a methodological contribution of this study.

By systematically testing theory-driven message variants on a population that mirrors real-world behavioral variance, this research provides empirical evidence for the effectiveness of different message framing strategies used in crisis communication response. This study offers a scalable, replicable, and data-driven framework for pre-testing and optimizing emergency messaging prior to real-world implementation.

Messages were designed using theoretical constructs from EPPM and TPB, focusing on fear appeals, efficacy cues, and social norms. To run the experiment, we built a digital twin of Miami-Dade County and included synthetic agents modeled on real demographic and environmental data. These agents were exposed to messages framed in different ways: fear-only, efficacy-only, norm-only, combined fear + efficacy, and combined fear + efficacy + norm, along with a neutral control message. This structure made it possible to compare how each type of framing influenced the likelihood of evacuation.

The following section presents the research questions tested in this study, describes how the digital twin was constructed, how synthetic agents were modeled, and how the MAF was applied to structure the message conditions and evaluate evacuation responses.

## 3 Methods

The following research questions were explored to evaluate the utility of using digital twins and agentic AI systems to test behavioral science theory:

RQ1: Do theory-based crisis communication messages (framed using EPPM and TPB constructs) increase evacuation compliance compared to neutral control messages?
RQ2: Which type of theory-based message framing—fear-only, efficacy-only, norm-only, combined fear + efficacy, or combined fear + efficacy + norm—is most effective at increasing evacuation compliance?

### 3.1 Study design

The current study used a cross-simulation, within-subjects (repeated measures) design using an agent population in a digital twin of Miami-Dade County, Florida. It is an ideal testbed for this project due to its high hurricane risk, large and diverse population, and national relevance in emergency response. These conditions provide opportunities to model, simulate, and validate crisis communication messages and strategies across different communities, ensuring realism and scalability for government agencies.

This study used a simulation approach with digital twins and synthetic agents. A major strength of this method is that it lets us carefully control conditions and test different scenarios without putting people at risk (Acharya et al., 2025; Botín-Sanabria et al., 2022; Pang et al., 2021; Pretel et al., 2024). The platform can support features like agent memory, social influence, and changing environmental conditions. However, in this experiment the primary interest is in a clean causal inference about message framing effects without confounds from message order and other temporal dynamics like warning fatigue. While the platform can support repeated messages over time, these temporal dynamics were outside the scope of this initial study and represent valuable directions for future research. Because some real-world factors were outside of the scope of this initial study and not included, and any results depend on the accuracy of the model and input data, any findings should be viewed as useful early evidence.

A total of sixteen message conditions were tested (Table 1). This included one control message without persuasive message framing. The other fifteen messages were based on EPPM and TPB theoretical constructs. There were five message categories with three messages per category that included: (1) Fear only, (2) Efficacy only, (3) Social norms only, (4) Fear and efficacy combined, and (5) Fear, efficacy, and social norms combined.

**Table 1:** Control and experimental messages by theoretical constructs.

Message variant id	Message category (construct)	Message content	Theoretical connection
C1	Control	A hurricane watch is currently in effect for your area. Stay tuned to local updates.	None (informational only)
F1	Fear-based	A hurricane watch is currently in effect for your area. Stay tuned to local updates. Storm surge will flood entire neighborhoods within hours. If you stay, emergency services may not be able to reach you.	Extended Parallel Process Model (EPPM)—emphasizes high threat and severity to encourage behavioral urgency.
F2	Fear-based	A hurricane watch is currently in effect for your area. Stay tuned to local updates. This hurricane is stronger than Hurricane Andrew, a Category 5 hurricane. Many lives were lost when people ignored evacuation orders—do not let history repeat itself.	EPPM—emphasizes perceived severity and historical risk to encourage behavioral urgency
F3	Fear-based	A hurricane watch is currently in effect for your area. Stay tuned to local updates. If you stay behind, you are risking your life and the lives of your children. This storm is deadly.	EPPM—appeals to perceived susceptibility and threat to others (social cognitive dimension)
E1	Efficacy-based	A hurricane watch is currently in effect for your area. Stay tuned to local updates. You can protect your family—evacuate now and head to the designated shelters. They are stocked and ready.	EPPM—enhances self-efficacy and response efficacy
E2	Efficacy-based	A hurricane watch is currently in effect for your area. Stay tuned to local updates. Evacuation routes are open and free. Police and emergency personnel are standing by to help you get out safely.	EPPM—enhances self-efficacy and response efficacy
E3	Efficacy-based	A hurricane watch is currently in effect for your area. Stay tuned to local updates. We’ve prepared shelters with food, power, and space for families and pets. You’re not alone—we have got you.	EPPM—increases confidence and promotes collective efficacy
M1	Combined fear-efficacy	A hurricane watch is currently in effect for your area. Stay tuned to local updates. This storm is deadly—but you can survive it by evacuating now. Shelters are ready and transportation is available.	EPPM—balances threat (fear) with self-efficacy (control) to produce adaptive behavior
M2	Combined Fear-Efficacy	A hurricane watch is currently in effect for your area. Stay tuned to local updates. Ignoring this storm could cost lives. But acting now can protect your loved ones—follow the evacuation guidance.	EPPM—combines fear appeal with self-efficacy, agency, and protection
M3	Combined fear-efficacy message	A hurricane watch is currently in effect for your area. Stay tuned to local updates. The danger is real, but so is your ability to act. Leave now, and you’ll be safe in a designated shelter.	EPPM—fuses high threat with actionable, low-barrier guidance to increase self-efficacy
T1	Subjective norm	A hurricane watch is currently in effect for your area. Stay tuned to local updates. Evacuation helps protect what matters most—your family and your home. Most of your community have already started evacuating the area. There are emergency personnel ready to assist you.	Theory of planned behavior—targets attitude subjective norm (do others expect me to evaluate)
T2	Subjective norm	A hurricane watch is currently in effect for your area. Stay tuned to local updates. Your neighbors are evacuating. Join them and stay safe.	Theory of planned behavior—targets attitude subjective norm (do others expect me to evaluate)
T3	Subjective norm	A hurricane watch is currently in effect for your area. Stay tuned to local updates. Do not be the last to evacuate. Everyone else is leaving.	Theory of planned behavior—targets attitude subjective norm (do others expect me to evaluate)
FEN1	Combined fear, efficacy, subjective norm	A hurricane watch is currently in effect for your area. Stay tuned to local updates. Staying behind could put you and your family in serious danger, floodwaters are expected to rise quickly. Most of your neighbors have already left for shelters, which are open and fully stocked. Join them now and keep your family safe.	EPPM/theory of planned behavior—integrates high threat (fear), strong efficacy, and subjective norm (peer action) to motivate adaptive evacuation behavior
FEN2	Combined fear, efficacy, subjective norm	A hurricane watch is currently in effect for your area. Stay tuned to local updates. This storm is unlike anything we have seen in years. Do not wait until it’s too late, evacuation routes are clear, and emergency teams are ready to help. Your community is counting on everyone to act quickly.	EPPM/theory of planned behavior—blends heightened threat (fear), actionable efficacy, and subjective norms (community expectations) to encourage evacuation
FEN3	Combined fear, efficacy, subjective norm	A hurricane watch is currently in effect for your area. Stay tuned to local updates. If you remain home, emergency responders may not be able to reach you during the worst of the storm. Most people in your area are already on their way to safety. Protect yourself and your loved ones by heading to the nearest shelter now.	EPPM/theory of planned behavior—combines risk (fear appeal), efficacy (clear action), and subjective norm (majority behavior) to prompt protective action

In each simulation run, agents received only a single message, and their internal states were reset afterward to eliminate any cumulative or order effects. A single message exposure serves as one input into the reasoning cycle at that point in time. Agents process the message against their internal beliefs, competing motivations, and personal characteristics, producing divergent intentions rather than a single threshold-based trigger.

However, in addition to embedding these behavioral constructs, it is essential to ensure that the agent population accurately reflects the demographics of Miami Dade County. We synthesized populations using publicly available datasets such as the American Community Survey (U.S. Census Bureau, 2025), the Behavioral Risk Factor Surveillance System (U.S. Centers for Disease Control and Prevention, 2025), and the Health Information National Trends Survey (National Cancer Institute, 2024). By matching demographic variables (age, race, and income) and health-related factors at the census tract level, each agent is grounded in a realistic socioeconomic context. With these realistic and autonomous agents that are embedded in a digital twin, crisis communicators can experiment with different message framings and quickly observe how populations might react, resulting in insights that are difficult to obtain from intuition alone.

#### 3.1.1 Digital twin

**Figure 2:**
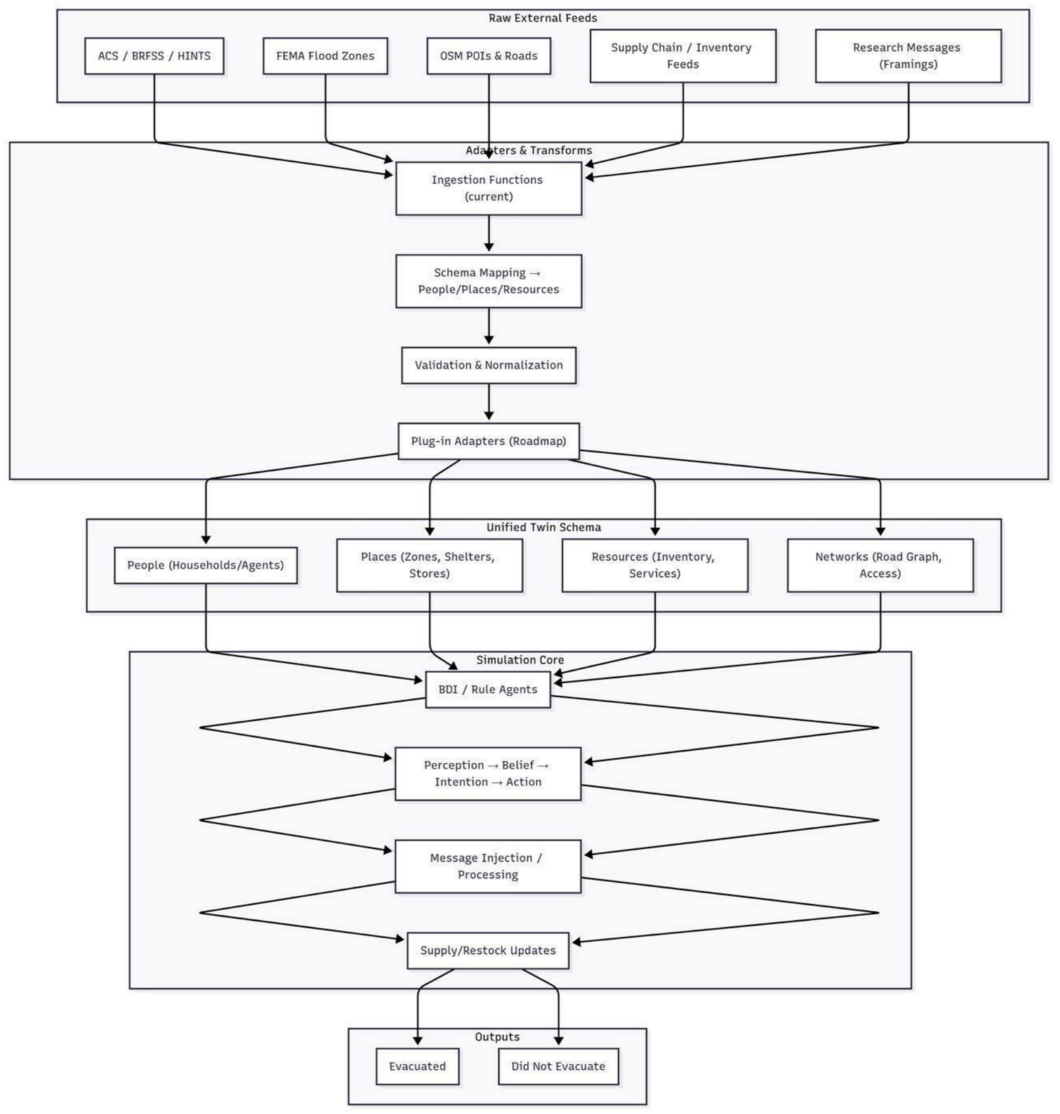
Dataflow and architecture of the digital twin simulation. The diagram illustrates how geospatial, demographic, and hazard layers generate the synthetic population, how messages enter the agent decision engine, and how evacuation outcomes are produced.

To set the parameters of the digital twin, four categories of data that anchor agent behavior in the Miami-Dade context are incorporated. Geographic layers define the ten spatial zones and road networks that directly represent the physical layout and transportation system. Demographic data populate each zone with agents whose age, income, education, and race or ethnicity distributions directly match local populations. Environmental risk layers apply FEMA flood zone classifications (FEMA, 2025) and historical hurricane experience to each zone to shape how prior exposure and location risk affect decision-making. See Figure 2 for further information on the data flow and digital twin architecture.

The digital twin was populated with 220 synthetic agents utilizing Bayesian-enhanced Multiple Imputation by Chained Equations to ensure representativeness and realism (Akande et al., 2017). Sample size per condition was estimated using parameters obtained from published literature and allowed for 95% power to detect a net difference with an estimated moderate effect size (Cohen’s *w* = 0.3) or larger between groups and accounted for multiple comparisons, which indicated a required number of 220 agents per condition (Vidrine et al., 2012). A total number of 3,550 total observations were collected across 15 experimental message framing conditions (three in each message framing category) and one control condition. There were 222 total agents populated into the digital twin simulation per condition, except for one fear-efficacy-norm condition that had 220 agents.

#### 3.1.2 Agent design

Each agent was assigned demographic and behavioral attributes that included age, education level, income, and theory-based behavioral drivers. A Belief-Desire-Intention (BDI) architecture (Archibald et al., 2024) was also embedded in the agents to allow agents to engage in goal-directed planning like real humans and unlike agent-based models. The agents make decisions through internal behavioral states and personal reasoning processes rather than fixed heuristics or probability tables. Each agent weighs beliefs, competing desires, personality traits, life circumstances, and the feasibility of action to form intentions that drive behavior. Demographic distribution directly aligned with the five-year estimates from the U. S. Census American Community Survey (U.S. Census Bureau, 2025) and was overlaid with the Federal Emergency Management Agency (FEMA) National Flood Hazard Layer maps (FEMA, 2025), Miami-Dade County shelter locations, and OpenStreetMap road network shapefiles (OpenStreetMap, n.d.). The agents maintained psychological states shaped by demographics, life circumstances such as immobility, household pets, financial or transportation barriers, and their prior experiences with hurricanes. Therefore, the agents perceived severity, susceptibility, self-efficacy, attitudes, subjective norms, and control that were initialized from each agent’s demographic and behavioral baseline. Once their internal states were established, decisions were generated through the BDI reasoning cycle informed by these attributes, rather than through static rule sets. Belief parameters such as perceived severity, susceptibility, self-efficacy, and subjective norm were informed by demographics and geospatial context, including FEMA flood zones, proximity to shelters, transportation access, and prior hurricane experience. These shaped baseline perceptions within the BDI reasoning cycle.

#### 3.1.3 Agent decision model specification

While agents reason internally through the BDI architecture, evacuation decisions are operationalized through an explicit and transparent decision rule to support interpretability and replicability. At the point of action selection, each agent computes an evacuation propensity based on the weighted influence of activated belief states and situational constraints. Specifically, perceived threat, perceived response efficacy, and perceived subjective norms increase evacuation propensity, while contextual constraints such as limited mobility, financial barriers, transportation access, and household obligations reduce the feasibility of evacuation.

Evacuation likelihood is represented conceptually as:


P(evacuate)=σ(αT+βE+γN−δC)

where T represents perceived threat, E represents perceived efficacy, N represents perceived subjective norms, C represents contextual constraints, and σ denotes a logistic transformation.

Within this framework, desires reflect agents’ competing goals (e.g., protecting family vs. avoiding disruption), and intentions represent the planned course of action that emerges when beliefs and desires are reconciled through the BDI reasoning process. These internal deliberations ultimately manifest in the evacuation propensity computed by the decision rule. While this formulation is presented as a probabilistic decision rule for clarity, the underlying implementation computes evacuation intent through weighted combinations of theory-driven belief activations and constraint checks with threshold-based action selection. Population-level variability emerges from heterogeneity in agent traits and repeated simulation runs rather than stochastic choice at the individual decision step.

Model parameters were not statistically estimated or fitted using historical evacuation outcome data. Instead, parameters were specified using a theory-consistent calibration approach informed by established findings from EPPM and TPB literature. Relative parameter weights were chosen to reflect empirical evidence that perceived threat and efficacy exert stronger influence on protective action than subjective norms alone (Tannenbaum et al., 2015; Witte and Allen, 2000), while contextual constraints reduce action feasibility (Morss et al., 2024; Rufat et al., 2024). All parameter values were held constant across simulation runs to maintain experimental control and ensure comparability across message framing conditions.

#### 3.1.4 Messaging simulation

This simulation was built in Python and implemented using the Mesa agent-based modeling framework (Masad and Kazil, 2015). Mesa allowed for structured agent scheduling and capture of agent actions, interactions, and aggregated outcomes throughout the simulation. The simulation was also enhanced and extended with external stimuli from the digital twin environment and theory-based behavioral drivers within the individual agents. The goal of the digital twin composition and simulation specifics was to provide a realistic environment for message testing that reflects actual data about the heterogeneity across the real population in Miami-Dade, FL.

Within this framework, the present study manipulated one independent variable, message framing. Agents were exposed to a series of crisis communication messages that varied by framing, where the content of the messages was systematically developed to reflect constructs from EPPM (Witte and Allen, 2000) and TPB (Tannenbaum et al., 2015). Each experimental (and the one control) condition involved the injection of one single evacuation message, followed by the observation of the key behavioral outcome and dependent variable, evacuation compliance. Agents processed message content through their BDI reasoning cycle, where theoretical constructs in the messages (see Table 1) activated corresponding belief states (e.g., perceived threat, efficacy, norms) already internalized in agent design based on each agent’s demographics, geospatial context, and prior hurricane experience. Message content was operationalized through structured, deterministic mappings between message components and EPPM/TPB constructs. Message content was pre-coded into theoretical constructs prior to simulation execution. Fear-framed messages deterministically increased perceived threat beliefs, efficacy-framed messages increased perceived response efficacy beliefs, and norm-framed messages increased subjective norm beliefs. Message text was not interpreted using natural language processing or language models. This deterministic mapping was predefined and applied consistently across all agents to ensure experimental control and eliminate variance from text interpretation, allowing the model to isolate the causal effects of specific message components.

This deterministic ingestion approach ensures reproducibility while the agentic component of the system is expressed in how agents subsequently update internal beliefs, form intentions, and select actions through their BDI reasoning process. This approach tests how message framing influences agent decision-making through their existing cognitive architecture rather than through hand-assigned numerical parameters or natural language processing methods. Fear-based messages emphasizing storm surge and danger engage agents’ threat-perception beliefs, while efficacy-based messages highlighting available shelters activate efficacy beliefs. Each agent weighs the message input against competing motivations, personal circumstances (financial barriers, mobility constraints, pet ownership, etc.), and geospatial context to form evacuation intentions, producing heterogeneous responses rather than predetermined mappings.

After each simulation run, we reset agent memory, erasing any memory of the previous message or agent responses to previous messages. All simulations were executed using fixed random seeds for each message condition, and each condition was replicated ten times with outcomes aggregated across runs to reduce stochastic variance. This design allows for completely independent, repeated testing across identical population conditions, eliminating order effects and other confounds common in field studies (Day et al., 2012; Duval and Hinz, 2019). Given identical initial conditions, parameter values, and random seeds, the simulation produces reproducible results, enabling independent replication of the reported findings. This approach enables a direct assessment of how variations in message framing influence behavioral responses at the individual and population level. This study did not simulate repeated or sequential messaging; each condition was treated as an independent, single exposure. This memory reset design, while enabling a clean causal inference about message framing effects, is similar to observing agents like survey respondents evaluating independent scenarios instead of cognitive actors accumulating information over time, a deliberate simplification for this proof-of-concept study that prioritizes experimental control over the ecological realism from continuous cognitive modeling. By resetting agent memory, we implemented a repeated-measures experimental design that isolates the effect of each message framing while controlling for individual agent characteristics. This is analogous to between-subjects experimental designs in behavioral research, where each participant sees only one condition. This approach allows for clean causal inference about message framing effects without confounds from message order, accumulation, or interaction effects. However, this design cannot capture temporal dynamics central to actual evacuation decision-making, including information accumulation, attitude crystallization, social influence propagation, or warning fatigue.

Behavioral data were collected at regular intervals throughout each run of the simulation, and each simulation was replicated ten times to ensure the robustness of the findings.

#### 3.1.5 Message assessment framework (MAF)

To guide the design and evaluation of crisis messages, we developed a *Message Assessment Framework (MAF)* that integrates EPPM, TPB and BDI architecture. The MAF operates on two levels: (1) message design, which classifies framings based on threat, efficacy, and social norms, and (2) agent decision-making, which embeds the same constructs into synthetic agents to shape behavioral responses (Figure 1). Table 2 summarizes the five components of the framework, including their theoretical basis and role in simulating evacuation decisions.

**Table 2:** Message assessment framework (MAF).

Component	Description	Supporting theory/reference
Behavioral theory	Core theoretical models provide the foundation for message framing and agent decision-making. This study used EPPM and TPB, but the framework can also incorporate models such as the Health Belief Model, Social Cognitive Theory, and Elaboration Likelihood Model.	EPPM; TPB; Health Belief Model; SCT; ELM
Message design (theory-based messaging)	Experimental messages were framed using constructs from EPPM (fear, efficacy) and TPB (subjective norms), tested individually and in combination. A neutral control was also included.	Witte and Allen (2000), Ajzen (2020), Tannenbaum et al. (2015)
Agent Design (theory-based population agents)	Agents were embedded with constructs aligned with message framings: perceived threat (severity/susceptibility), efficacy (response/self-efficacy), and subjective norms. These shaped how agents processed messages and made evacuation decisions.	Morss et al. (2024), DiCarlo and Berglund (2020)
Belief–desire–intention (BDI) architecture	BDI architecture allowed agents to engage in goal-directed reasoning and planning, beyond simple “if–then” rules, improving realism in decision-making.	Archibald et al. (2024)
Behavioral outputs (evacuation vs. non-evacuation)	Outcomes depended on agents’ internal states and message framing. For example, high threat + high efficacy increased evacuation, while high threat + low efficacy led to fear control or inaction.	Schmidt-Colberg et al. (2024), Morss et al. (2024)

Williams (2023) supports the combination of theoretical models to improve the ability to explain and predict human behavior in comparison to using just a single theoretical framework. In the Message Assessment Framework, we have included the following multiple dimensions: (1) Emotional factors such as fear and perceived threat from EPPM; (2) Subjective norms from TPB that include an individual’s beliefs of the expectations of others to act in a particular way; and (3) Intentional reasoning where autonomous agents have goals and decide on actions based on BDI.

The integration of multi-theoretical frameworks in message design, specifically EPPM and TPB, contributes to determining whether an agent enacts protective behaviors and acts based on social norms, as well as the agent’s beliefs, desires, and intentions (Figure 1). The framework offers flexibility in testing different combinations of social and behavioral theories. This framework also enables emergency personnel to evaluate emergency evacuation messaging and insights as to the message framing that would be most effective in achieving desired behavioral outcomes.

## 4 Results

Descriptive statistics were first calculated to provide an overview of agent responses across all message conditions, facilitate comparison across conditions, and inform subsequent hypothesis testing. For each experimental condition and the control group, we calculated the total number and percentage of agents who chose to evacuate. These summary statistics included frequencies, proportions, and the mean evacuation rate by condition. This initial step allowed for visualization of the evacuation decisions to identify any preliminary patterns of differences between message types prior to inferential analysis.

Next, evacuation compliance was analyzed in two stages. In the first step, we ran a chi-square test to see if evacuation rates were different across the 16 types of messages. This gave us an overall sense of whether the kind of message influenced people’s decisions to evacuate. After finding a clear difference, we conducted a logistic regression to measure how each message type compared to the neutral control message. Logistic regression was selected because it is well suited for binary outcomes (evacuate vs. not evacuate) and produces odds ratios with confidence intervals, allowing for direct comparison of the relative effectiveness of the different message strategies.

To test the first research question to see if theory-based messages led to different evacuation rates compared to neutral control messages, a chi-squared test of independence was conducted. Evacuation rates differed significantly across the 16 message framing conditions (1 control, 3 fear-only, 3 efficacy-only, 3 norms-only, 3 fear + efficacy, 3 fear + efficacy + norms) (χ^2^ = 323.2, df = 15, *N* = 3,550, *p* < 0.01; see Table 3). The chi-square test of independence was included as an overall test to determine whether evacuation compliance varied systematically across the 16 message conditions (Table 4). The significant result showed that evacuation compliance varied across message conditions, which justified moving forward with pairwise comparisons and regression modeling to determine which specific framings drove these differences.

**Table 3:** Evacuation rates by message condition.

Condition	# Evacuated	# Not evacuated	Evacuation %
Control	2	220	0.9% evacuated.
Fear 1	2	220	0.9% evacuated.
Fear 2	2	220	0.9% evacuated.
Fear 3	2	220	0.9% evacuated.
Efficacy 1	3	219	1.4% evacuated.
Efficacy 2	3	219	1.4% evacuated.
Efficacy 3	2	220	0.9% evacuated.
Subj. norm 1	2	220	0.9% evacuated.
Subj. norm 2	2	220	0.9% evacuated.
Subj. norm 3	2	220	0.9% evacuated.
Fear-efficacy 1	26	196	11.7% evacuated.
Fear-efficacy 2	44	178	19.8% evacuated.
Fear-efficacy 3	12	210	5.4% evacuated.
Fear-efficacy-norm 1	28	194	12.6% evacuated.
Fear-efficacy-norm 2	48	174	21.6% evacuated.
Fear-efficacy-norm 3	26	194	11.8% evacuated.
Total	206	3,344	

**Table 4:** Adjusted standardized residuals for chi-square results by condition.

Condition	Adj. std. residual
Control	−3.23**
Fear 1	−3.23**
Fear 2	−3.23**
Fear 3	−3.23**
Efficacy 1	−2.93
Efficacy 2	−2.93
Efficacy 3	−3.23**
Subj. norm 1	−3.23**
Subj. norm 2	−3.23**
Subj. norm 3	−3.23**
Fear-efficacy 1	3.89**
Fear-efficacy 2	9.23**
Fear-efficacy 3	−0.26
Fear-efficacy-norm 1	4.48**
Fear-efficacy-norm 2	10.41**
Fear-efficacy-norm 3	3.94**

** Statistically significant at *p* < 0.01. Used Bonferroni correction for multiple comparisons.

The highest evacuation compliance was seen with the fear–efficacy–norm messages (15.4%), followed by the fear–efficacy messages (12.3%), compared with only 0.9% in the control group. Only the multi-component messages led to significant increases in compliance after adjusting for multiple comparisons, while the single-component messages (fear-only, efficacy-only, norm-only) showed no meaningful differences from control (Table 5).

**Table 5:** Evacuation compliance rates by message framing.

Message frame	Number of agents	Compliance rate (%)
Control	222	0.9%
Fear only (average of 3 messages)	666	0.9%
Efficacy only (average of 3 messages)	666	1.2%
Subjective norm only (avg. of 3 messages)	666	0.9%
Fear + efficacy (avg. of 3 messages)	666	12.3%
Fear + efficacy + norm (avg. of 3 messages)	664	15.4%

Rates are a percentage of agents who evacuated.

To test the second research question, which explored which message frame led to the highest evacuation compliance, a logistic regression analysis was performed. The results showed that the odds of evacuation were about 15 times higher for fear–efficacy messages and nearly 20 times higher for fear–efficacy–norm messages relative to the control message (see Table 6). In contrast, the single-component conditions did not produce significant effects. While the odds ratio for fear–efficacy–norm messages was greater than that for fear–efficacy alone, their overlapping confidence intervals indicate that the difference was not statistically reliable. These findings suggest the effectiveness of fear and efficacy in increasing compliance, whereas the added impact of normative messaging cues remains uncertain.

**Table 6:** Logistic regression results for evacuation compliance by message framing.

Message framing vs. neutral	OR	95% CI	*p*-value
Control	0.0091	[0.002, 0.003]	*p* < 0.001 **
Fear-based	1.00	[0.23, 6.86]	*p* > 0.05
Efficacy-based	1.34	[0.33, 8.90]	*p* > 0.05
Subjective norm-based	1.00	[0.23, 6.86]	*p* > 0.05
Combined fear + efficacy	15.45	[4.82, 94.34]	*p* < 0.001 **
Fear + efficacy + norm	19.98	[6.26, 121.72]	*p* < 0.001 **

** Statistically significant at *p* < 0.01. *Reference category: neutral message. Odds ratios (OR) greater than 1 indicate higher odds of evacuation relative to the neutral framing.

## 5 Discussion

The most notable observation is that while the fear + efficacy + norm message condition produced the highest odds ratio for evacuation compliance (*OR* = 19.98, approximately 20 times higher than control), its confidence interval overlapped with that of the fear + efficacy condition (*OR* = 15.45). This indicates that the added benefit of the two multi-component strategies were not statistically distinguishable in this sample. However, the pattern supports TPB’s prediction that norms enhance compliance when combined with threat and efficacy, as the fear + efficacy + norm condition achieved the highest compliance rate (15.4%) compared to all other message framing conditions. Norm-only messages showed minimal effects (0.9%), consistent with literature suggesting that norms work best when paired with other motivational cues (Tabatabaei et al., 2025). Future work with larger sample sizes, a wider range of message exemplars, and other types of disaster events may help determine whether normative cues provide an additive effect beyond fear and efficacy or whether their influence is already captured through the combined framing.

Messages that combined fear and efficacy also had a strong impact. This finding is consistent with past research (with human participants instead of simulated agents) showing that people are more likely to act when they understand the threat and also know what steps to take (Ajzen, 2020; Tannenbaum et al., 2015; Witte and Allen, 2000). One actionable insight for emergency planners and crisis communicators is to combine fear and efficacy messages, given that this study adds to previous evidence by demonstrating the effectiveness of fear and efficacy messages in a simulated environment.

The findings have implications that can inform natural disaster response and evacuation compliance, panic reduction, crisis communication, health communication, and behavior change theory by informing the design of more effective crisis messaging strategies, with the potential to positively enhance behavioral outcomes. These findings should be interpreted in the context of prior research, which shows that evacuation behavior is complex and multi-dimensional, involving decisions about whether to evacuate, when to leave, where to go, how to travel, and which routes to take (Collins et al., 2024; Sadri et al., 2021). Additional response and messaging parameters can also be incorporated into future experiments to optimize response across different disaster scenarios or localities.

There are also implications for using Agentic AI to test behavioral theory. This study represents a step toward integrating empirically validated behavioral theory with agentic AI and digital twin simulation technologies in the context of disaster response for pre-testing communication interventions. The approach is designed to complement, rather than replace, traditional field studies, enabling practitioners to identify which message framing strategies are likely to yield the highest compliance. Ultimately, the goal is to support a transition from intuition-based to evidence-driven crisis communication. The implications for future research are significant, influencing the potential for further refinement of agent models, expansion to additional behaviors and variables, incorporation of additional types of data and behavioral theories, and application to a broader range of crisis scenarios.

### 5.1 Limitations and future research

The study focused on three message elements that include fear, efficacy, and norms, while holding other factors constant, in a hurricane evacuation scenario. Both the fear + efficacy and fear + efficacy + norm conditions increased evacuation compliance compared with fear-only, but the results for those two combined conditions were not clearly different from each other.

The modest additive effect of norm-framed messages likely reflects a fundamental design choice in our experimental approach. In this study, social norms were operationalized as message content emphasizing community expectations and peer behavior (e.g., “your neighbors are evacuating”), not as dynamic peer-to-peer influence through social network interactions. Agents did not observe or influence each other’s evacuation decisions in real-time. This design choice allowed us to isolate the effect of normative message framing while holding social interaction constant, ensuring that observed differences in evacuation behavior could be attributed to message content rather than to dynamic peer influence. In real evacuations, individuals often observe neighbors and other peers preparing to leave and adjust their own decisions accordingly, dynamics that were intentionally held constant in this study to maintain experimental control. Future work could relax this constraint by integrating social network modeling to allow agents to observe and respond to peers’ evacuation decisions dynamically, which would likely amplify the impact of normative messaging and enable the examination of how message framing and social influence interact. Additionally, to test the question about the added effect of norms directly, future studies should compare messages with and without norms using larger samples to provide stronger statistical evidence. Researchers should also try a wider range of message types and include other important factors, such as source credibility and repeated exposure over time. To make the results more generalizable, testing should be conducted across different populations, cultural groups, and hazard types. This framework can also extend to wildfires, floods, and infectious disease outbreaks by integrating relevant hazard and disease spread models while maintaining theory-based communication structures. While this study emphasizes theory-driven calibration and controlled simulation experiments, future work should validate and refine agent decision parameters using observed evacuation data from historical hurricane events and post-event behavioral studies.

The choice of 220 agents per condition was based on *a priori* power analysis but may not fully capture Miami-Dade’s complete demographic diversity. Scaling to larger populations could reveal finer-grained effects and better represent heterogeneity, potentially resolving subtle differences between conditions (i.e., the overlapping confidence intervals between fear + efficacy and fear + efficacy + norm conditions). Future validations could increase agent count without the risk of biasing the results, as the model accounts for individual variability rather than averaging behavior across populations. Future research should also examine computational scalability by systematically testing simulation runtime as a function of agent population size, which would inform the feasibility of scaling this framework for operational deployment. Advancing toward real-time emergency analysis capabilities will require careful testing and collaboration with emergency management practitioners to expand utility and ensure the system meets practical needs to integrate effectively with existing decision-making workflows.

Additionally, the Message Assessment Framework allows for flexibility in expanding to other behavioral theories. Future research should include the evaluation of additional behavioral theories in the context of crisis communication to provide expanded options for crisis messaging. Future models should account for linguistic and cultural diversity by assigning agents language preferences (e.g., English/Spanish communication in Miami-Dade County) and culturally grounded behavior patterns, including trust, norms and message receptivity.

To improve the simulation and make the model closer to real behavior, details about how people influence each other through social networks, how decision styles differ across individuals, and how attitudes change between receiving a message and acting should be included, in addition to other factors including alert or warning fatigue (Sutton and Wood, 2025). These improvements should be checked against field studies with real people to make sure predictions match reality. By doing this, future research can build stronger evidence for designing effective crisis messages. The current research involved the development of digital twin technology, the foundation of which can evolve to incorporate enhancements such as new concepts from additional behavioral theories to continue exploring this space.

The major focus of the current study was to isolate and test the independent effects of single message exposures to establish a proof-of-concept for theory-driven digital twin simulation. The controlled experimental design testing one message at a time with memory reset between conditions enabled a clean comparison across message framing but represents a fundamental trade-off between experimental control and ecological validity. While this approach allows for rigorous causal inference about message framing effects, it does not attempt to model the full complexity of multi-day hurricane events with repeated communications, evolving information landscapes, and dynamic peer influence that characterize real-world evacuation scenarios.

Future research should build on this foundation in two key directions. First, extending the simulation to model repeated and sequential messaging over time would examine reinforcement dynamics and how warning fatigue or other cumulative effects shape evacuation decisions. Second, validating simulation outputs against historical evacuation data from events such as real hurricane situations would establish the model’s predictive accuracy and help refine agent behavioral parameters to better match observed human responses. Comparing simulated compliance rates by demographic segment and geographic zone to documented historical patterns would strengthen confidence in the model’s ability to forecast real-world outcomes while identifying areas where agent reasoning may need enhancement.

## 6 Conclusion

This study provides a prototype and foundation for future work using digital twin and agentic AI technology for behavioral science and crisis communication research. By comparing agent response to different messages, the approach provides scalable simulations in realistic environments that test the effects of theory-based (EPPM, TPB) messaging on evacuation behavior in dynamic, high-risk scenarios. Unlike traditional agent-based models that rely on heuristics or rule-based logic, our approach leverages the BDI architecture, enabling agents to reason and adapt in response to message framing, social context, and environmental cues. This enhances the ecological validity of simulated behavioral outcomes, particularly in complex public safety scenarios such as hurricane evacuation (Chapuis et al., 2022; Pretel et al., 2024).

To systematically evaluate the effects of message design, we developed the Message Assessment Framework (MAF), which is a configurable tool that enables rapid, low-risk experimentation, tracking, and comparison of theory-based messages within digital twin environments prior to field studies or real-world deployment. MAF provides a structured tool to assess how changes in message framing influence individual agent decisions and population-level outcomes over time. In the current study, MAF classified and evaluated crisis response messages that varied in their emphasis on threat, efficacy, and social norms cues, as well as combinations of all three.

Our findings show that messages combining high-threat and high-efficacy appeals (aligned with EPPM) and those emphasizing social norms and behavioral control (aligned with TPB) produced significantly higher rates of simulated evacuation compliance compared to single-cue or the neutral control message. These results advance the literature in applying artificial intelligence to behavioral science by demonstrating that, in a synthetic population grounded in real-world data, combining threat, efficacy, and social norm appeals are more effective approaches in a hurricane evacuation situation than messaging strategies that rely on fear, efficacy, or social norm cues alone.

This study establishes a foundation for using digital twins and agentic AI for behavioral science and offers a powerful and scalable method for pre-testing and optimizing crisis communication strategies before implementing them in the real world. The integration of the MAF within a realistic digital twin addresses a critical gap in the message effects literature and represents a significant step in bridging behavioral theory and AI-driven experimentation by allowing researchers to test many message variants across demographically realistic populations in ways that are both fast and empirically grounded. Future work can build on this research by expanding the framework to include additional behavioral theories, message types, and crisis contexts, which will further enhance the capacity for data-driven decision-making in emergency communication and disaster response. Looking ahead, this digital twin framework could be integrated into real-time emergency management systems as a decision-support tool. As digital twins increasingly incorporate real-time data, this framework could support emergency managers by rapidly testing alternative message framings to forecast compliance during unfolding events. Although this would require robust calibration and computing resources, our results demonstrate the potential value of such an approach for adaptive, data-driven crisis communication.

## Data Availability

The data supporting the conclusions of this study are not publicly available due to internal MITRE restrictions. De‑identified data and supporting materials may be made available from the corresponding author upon reasonable request and with MITRE approval.

## References

[ref1] Acharya D. B. Kuppan K. Divya B. (2025). Agentic AI: autonomous intelligence for complex goals - a comprehensive survey. IEEE Access 13, 18912–18936. doi: 10.1109/ACCESS.2025.3532852

[ref2] Adjei E. Benedict B. Murray-Tuite P. Lee S. Ukkusuri S. Ge Y. (2022). Effects of risk perception and perceived certainty on evacuate/stay decisions. Int. J. Disaster Risk Reduct. 80:103247. doi: 10.1016/j.ijdrr.2022.103247

[ref3] Ajzen J. (2020). The theory of planned behavior: frequently asked questions. Hum. Behav. Emerg. Technol. 21, 314–324. doi: 10.1002/hbe2.195

[ref4] Akande O. Li F. Reiter J. (2017). An empirical comparison of multiple imputation methods for categorical data. Am. Stat. 71, 162–170. doi: 10.1080/00031305.2016.1277158

[ref5] Archibald B. Calder M. Sevegnani M. Xu M. (2024). Quantitative modelling and analysis of BDI agents. Softw. Syst. Model. 23, 343–367. doi: 10.1007/s10270-023-01121-5

[ref6] Armitage C. J. Conner M. (2001). Efficacy of the theory of planned behavior: a meta-analytic review. Br. J. Soc. Psychol. 40, 471–499. doi: 10.1348/014466601164939, 11795063

[ref7] Bigsby E. Albarracin D. (2022). Self- and response efficacy information in fear appeals: a meta-analysis. J. Commun. 72, 241–263. doi: 10.1093/joc/jqab048, 40963740 PMC12437762

[ref8] Bilal S. Zaatour W. Alonso Otano Y. Saha A. Newcomb K. Kim S. . (2025). CitySEIRCast: an agent-based city digital twin for pandemic analysis and simulation. Complex Intell. Syst. 11:83. doi: 10.1007/s40747-024-01683-x

[ref9] Botín-Sanabria D. M. Mihaita A.-S. Peimbert-García R. E. Ramírez-Moreno M. A. Ramírez-Mendoza R. A. Lozoya-Santos J. d. J. (2022). Digital twin technology challenges and applications: a comprehensive review. Remote Sens 14:1335. doi: 10.3390/rs14061335

[ref10] Chapuis K. Tailandier P. Drogoul A. (2022). Generation of synthetic populations in social simulations: a review of methods and practices. J. Artif. Soc. Soc. Simul. 25:6. doi: 10.18564/jasss.4762

[ref11] Collins J. Dunn E. A. Jones R. J. Pollen A. Rao N. R. Murphy S. . (2024). Hurricane risk perceptions and evacuation decision-making in the postvaccine era of COVID-19 in U.S. coastal states impacted by North Atlantic hurricanes. Weather Clim. Soc. 16, 51–65. doi: 10.1175/WCAS-D-23-0003.1

[ref12] Collins J. Ersing R. Polen A. Saunders M. Senkbeil J. (2018). The effects of social connections on evacuation decision making during hurricane Irma. Weather Clim. Soc. 10, 459–469. doi: 10.1175/WCAS-D-17-0119.1

[ref13] Croatti A. Gabellini M. Montagna S. Ricci A. (2020). On the integration of agents and digital twins in healthcare. J. Med. Syst. 44:161. doi: 10.1007/s10916-020-01623-5, 32748066 PMC7399680

[ref14] Day B. Bateman I. Carson R. Dupont D. Louviere J. Morimoto S. . (2012). Ordering effects and choice set awareness in repeat-response stated preference studies. J. Environ. Econ. Manag. 63, 73–91. doi: 10.1016/j.jeem.2011.09.001

[ref15] Dembski F. Wössner U. Letzgus M. Ruddat M. Yamu C. (2020). Urban digital twins for smart cities and citizens: the case study of Herrenberg, Germany. Sustainability 12:2307. doi: 10.3390/su12062307

[ref16] DiCarlo M. Berglund E. (2020). Use of social media to seek and provide help in hurricanes Florence and Michael. Smart Cities 3, 1187–1218. doi: 10.3390/smartcities3040059

[ref17] Duval S. Hinz T. (2019). Different order, different results? The effects of dimension order in factorial survey experiments. Field Methods 32:827. doi: 10.1177/1525822X19886827

[ref18] Edwards D. J. (2021). Ensuring effective public health communication: insights and modeling efforts from theories of behavioral economics, heuristics, and behavioral analysis for decision making under risk. Front. Psychol. 12:715159. doi: 10.3389/fpsyg.2021.715159, 34721162 PMC8548420

[ref19] FEMA 2025 Flood data viewers and geospatial data Available online at: https://www.fema.gov/flood-maps/national-flood-hazard-layer (Accessed September 9, 2025).

[ref20] Galuzin V. Galitskaya A. Grachev S. Larukhin V. Novichkov D. Skobelev P. . (2022). Autonomous digital twin of enterprise: method and toolset for knowledge-based multi-agent adaptive management of tasks and resources in real time. Mathematics 10:1662. doi: 10.3390/math10101662

[ref21] Ghaffarian S. (2025). Rethinking digital twin: introducing digital risk twin for disaster risk management. NPJ Nat. Hazards 5:135. doi: 10.1038/s44304-025-00135-x

[ref22] Islam K. Akhther N. Seeger M. W. (2023). Variability in media content of public health outbreak coverage: a crisis communication approach. Commun. Stud. 74, 113–130. doi: 10.1080/10510974.2023.2183516

[ref23] Kim M. Kim S. Jeon S. (2024). One in three or three in one: integrating three competing theoretical models (TPB, VIP, and PADM) to explain the intentions to act/actions against climate change. Heliyon 10:e39337. doi: 10.1016/j.heliyon.2024.e39337, 39553624 PMC11565018

[ref24] Logan M. Bradley B. M. Chen B. Kruger J. Van Meter J. Paetznick B. . (2022). A policy analysis of preparedness for hurricane evacuations in the United States, 1990 to 2019: implementation in coastal states. Health Secur. 20, 65–73. doi: 10.1089/hs.2021.0125, 34935495 PMC10036075

[ref25] Masad D. Kazil J. (2015). Mesa: An Agent-Based Modeling Framework. Austin, TX: SciPy.

[ref26] Morss R. Cuite C. Demuth J. (2024). What predicts hurricane evacuation decisions? The importance of efficacy beliefs, risk perceptions, and other factors. Nat. Hazards 1:24. doi: 10.1038/s44304-024-00025-8

[ref27] Munro M. Gore R. J. Lynch C. J. Hastings Y. D. Reinhold A. M. (2025). Enhancing risk and crisis communication with computational methods: a systematic literature review. Risk Anal. 45, 1683–1697. doi: 10.1111/risa.17690, 39676035 PMC12396938

[ref28] Naroozi Masir M. Tarrahi M. Fathian Dastgerdi Z. Rahimi M. (2023). Investigating the factors related to protective behaviors against COVID-19 in healthcare workers: application of extended parallel process model. Health Sci. Rep. 6:e1778. doi: 10.1002/hsr2.1778, 38125278 PMC10731120

[ref29] National Cancer Institute. (2024). Health information National Trends Survey. Available online at: https://hints.cancer.gov/ (Accessed September 13, 2025).

[ref30] OpenStreetMap (n.d.) OpenStreetMap. Available online at: openstreetmap.org/ (Accessed September 9, 2025).

[ref31] Ort A. Fahr A. (2018). Using efficacy cues in persuasive health communication is more effective than employing threats - an experimental study of a vaccination intervention against Ebola. Br. J. Health Psychol. 23, 665–684. doi: 10.1111/bjhp.12310, 29635864

[ref32] Pang J. Huang Y. Xie Z. Li J. Cai Z. (2021). Collaborative city digital twin for the COVID-19 pandemic: a federated learning solution. Tsinghua Sci. Technol. 26, 759–771. doi: 10.26599/TST.2021.9010026

[ref33] Penverne Y. Martinez C. Cellier N. Pehlivan C. Jenvrin J. Savary D. . (2024). A simulation-based digital twin approach to assessing the organization of response to emergency calls. NPJ Digit. Med. 7:392. doi: 10.1038/s41746-024-01392-2 PMC11688439 39741218

[ref34] Pretel E. Moya A. Navarro E. López-Jaquero V. González P. (2024). Analysing the synergies between multi-agent systems and digital twins: a systems literature review. Inf. Softw. Technol. 174:107503. doi: 10.1016/j.infsof.2024.107503

[ref35] Riaz K. McAfee M. Gharbia S. S. (2023). Management of climate resilience: exploring the potential of digital twin technology, 3D city modelling, and early warning systems. Sensors 23, 26–59. doi: 10.3390/s23052659, 36904867 PMC10007107

[ref36] Rufat S. Comby E. Lhomme S. Santoni V. (2024). Context matters when evacuating large cities: shifting the focus from individual characteristics to location and social vulnerability. Environ. Sci. Pol. 162, 1–10. doi: 10.1016/j.envsci.2024.103925

[ref37] Sadri A. Ukkusuri S. Ahmed M. (2021). Review of social influence in crisis communications and evacuation decision-making. Transp. Res. Interdiscip. Perspect. 9, 1–12. doi: 10.1016/j.trip.2021.100325

[ref38] Schmidt-Colberg A. Teigler S. Meyer R. Kruse A., 2024. Towards modelling human behaviour and warning message informativity in large-scale event evacuation, Berlin. Proceedings of the 23rd International Conference on Modeling & Applied Simulation.

[ref39] Seeger M. W. (2006). Best practices in crisis communication: an expert panel process. J. Appl. Commun. Res. 34, 232–244. doi: 10.1080/00909880600769944

[ref40] Shultz J. M. Kossin J. P. Hertelendy A. Burkle F. Fugate C. Sherman R. . (2020). Mitigating the twin threats of climate-driven Atlantic hurricanes and COVID-19 transmission. Disaster Med. Public Health Prep. 14, 494–503. doi: 10.1017/dmp.2020.243, 32660664 PMC7387761

[ref41] Siegenthaler P. Ort A. Fahr A. (2021). The influence of valence shifts in fear appeals on mesage processing and behavioral intentions: a moderated mediation model. PLoS One 16:e0255113. doi: 10.1371/journal.pone.0255113, 34473710 PMC8412313

[ref42] Srivastava S. K. Mishra A. Singh S. Jaiswal D. (2023). Household food waste and theory of planned behavior: a systematic review and meta-analysis. Environ. Sci. Pollut. Res. 30, 97645–97659. doi: 10.1007/s11356-023-29141-0, 37594711

[ref43] Sutton J. Wood M. M. (2025). Opting out: over-alerting and warning fatigue in the era of wireless emergency alerts. J. Contingencies Crisis Manage. 33:e70076. doi: 10.1111/1468-5973.70076

[ref44] Tabatabaei S. Shahesmaeilinejad A. Moghaddam S. Davarani F. (2025). Determinants of disaster preparedness behaviors based on the theory of planned behavior among residents of Kerman, Iran: a cross-sectional study. Open Public Health J. 18:e18749445382578. doi: 10.2174/0118749445382578250328055314

[ref45] Taghikhah F. Jabbari A. Desouza K. C. Malik A. Khorshidi H. A. (2025). Understanding delayed diabetes diagnosis: an agent-based model of health-seeking behavior. Med. Decis. Mak. 45, 399–425. doi: 10.1177/0272989x251326908, 40183324 PMC11992636

[ref46] Tannenbaum M. B. Hepler J. Zimmerman R. S. Saul L. Jacobs S. Wilson K. . (2015). Appealing to fear: a meta-analysis of fear appeal effectiveness and theories. Psychol. Bull. 141, 1178–1204. doi: 10.1037/a0039729, 26501228 PMC5789790

[ref47] Taylor N. Healey E. Morrow A. Greening S. Wakefield C. E. Warwick L. . (2020). Aligning intuition and theory: enhancing the replicability of behavior change interventions in cancer genetics. Implement. Sci. Commun. 1:90. doi: 10.1186/s43058-020-00054-0 33073243 PMC7557091

[ref48] Taylor N. McKay S. Long J. C. Gaff C. North K. Braithwaite J. . (2023). Aligning intuition and theory: a novel approach to identifying the determinants of behaviours necessary to support implementation of evidence into practice. Implement. Sci. 18:29. doi: 10.1186/s13012-023-01284-1, 37475088 PMC10360252

[ref49] Tsoy D. (2022). Impact of social media, extended parallel process model and stay-at-home intentions during COVID-19. Sustainability 14:7192. doi: 10.3390/su14127192

[ref50] U.S. Census Bureau (2025) American community survey (ACS) Available online at: https://www.census.gov/programs-surveys/acs/ (Accessed September 9, 2025).

[ref51] U.S. Centers for Disease Control and Prevention, 2025. Behavioral risk factor surveillance system. [Online] Available online at: https://www.cdc.gov/brfss/index.html (Accessed September 13, 2025).

[ref52] Vidrine D. J. Fletcher F. E. Danysh H. E. (2012). A randomized controlled trial to assess the efficacy of an interactive mobile messaging intervention for underserved smokers. BMC Public Health 12:696. doi: 10.1186/1471-2458-12-696 22920991 PMC3585470

[ref53] Vos S. C. Buckner M. M. (2016). Social media messages in an emerging health crisis: tweeting bird flu. J. Health Commun. 21, 301–308. doi: 10.1080/10810730.2015.1064495, 26192209

[ref54] Wang L. Jiang Y. Pan L. Ji J. Xu A. (2023). Research on household emergency supplies storage from the theory of planned behavior and intention-behavior gap in the context of COVID-19. Frontiers 13:1069843. doi: 10.3389/fpsyg.2022.1069843 PMC9885126 36726511

[ref55] Wang Y. Kyriakidis M. Dang V. N. (2021). Incorporating human factors in emergency evacuation – an overview of behavioral factors and models. Int. J. Disaster Risk Reduct. 60:102254. doi: 10.1016/j.ijdrr.2021.102254

[ref56] Williams D. (2023). A meta-theoretical framework for organizing and integrating theory and research on motivation for health-related behavior. Front. Psychol. 14:1130813. doi: 10.3389/fpsyg.2023.1130813, 36910809 PMC9995609

[ref57] Witte K. (1992). Putting the fear back into fear appeals: the extended parallel process model. Commun. Monogr. 59, 329–349. doi: 10.1080/03637759209376276

[ref58] Witte K. Allen M. (2000). A meta-analysis of fear appeals: implications for effective public health campaigns. Health Educ. Behav. 27, 591–615. doi: 10.1177/109019810002700506, 11009129

[ref59] Wong-Parodi G. Garfin D. R. (2022). Priming close social contact protective behaviors enhances protective social norms perceptions, protection views, and self-protective behaviors during disasters. Int. J. Disaster Risk Reduct. 80:103135. doi: 10.1016/j.ijdrr.2022.103135, 35784266 PMC9233988

[ref60] Woo Yoo C. Lee J. Yoo C. Xiao N. (2021). Coping behaviors in short message service (SMS)-based disaster alert systems: from the lens of protection motivation theory as elaboration likelihood. Inf. Manag. 58:103454. doi: 10.1016/j.im.2021.103454

[ref61] Wright L. Davidson L. (2020). How to tell the difference between a model and a digital twin. Adv. Model. Simul. Eng. Sci. 7:147. doi: 10.1186/s40323-020-00147-4

